# Cardiac Outlines

**Published:** 1893-06

**Authors:** 


*Cardiac Outlines.* By WILLIAM EWART, M.D., F.R.C.P. Pp. x., 165. London: BAILLIÈRE, TYNDALL AND COX. 1892.

This will be found a very useful manual both for practitioners and also for students and teachers. More especially, perhaps, it will meet the wants of the two latter. Nothing is more important for the student than that he should early learn and systematically carry out a methodical and thorough examination of the heart. A complete training in this is an invaluable method of inculcating in him methods of accuracy and precision in his daily work, and of impressing upon him the necessity of careful investigation as the first step to a sound diagnosis. Properly grounded in this manner he will be ready to intelligently deal with the problems presented by any particular case. The student who has carefully followed out the teaching of this book, and accustomed himself to the practical application of the methods detailed in it, will possess such a training. The work is throughout illustrated by well-executed outlines. We do not think that it is advisable for the pleximeter to be used until the ordinary method of percussion has been thoroughly mastered; and this, in our opinion, is, of all the methods of physical diagnosis, the most difficult to acquire. We are disposed to think that a short description of the use of the sphygmograph and cardiograph in clinical practice might be added with advantage to future editions of this excellent little book.

